# Electroacupuncture Reduces Weight Gain Induced by Rosiglitazone through PPAR*γ* and Leptin Receptor in CNS

**DOI:** 10.1155/2016/8098561

**Published:** 2016-01-20

**Authors:** Xinyue Jing, Chen Ou, Hui Chen, Tianlin Wang, Bin Xu, Shengfeng Lu, Bing-Mei Zhu

**Affiliations:** ^1^Key Laboratory of Acupuncture and Medicine Research of Ministry of Education, Nanjing University of Chinese Medicine, Nanjing 210023, China; ^2^Jiangxi University of Traditional Chinese Medicine, Nanchang 330004, China

## Abstract

We investigate the effect of electroacupuncture (EA) on protecting the weight gain side effect of rosiglitazone (RSG) in type 2 diabetes mellitus (T2DM) rats and its possible mechanism in central nervous system (CNS). Our study showed that RSG (5 mg/kg) significantly increased the body weight and food intake of the T2DM rats. After six-week treatment with RSG combined with EA, body weight, food intake, and the ratio of IWAT to body weight decreased significantly, whereas the ratio of BAT to body weight increased markedly. HE staining indicated that the T2DM-RSG rats had increased size of adipocytes in their IWAT, but EA treatment reduced the size of adipocytes. EA effectively reduced the lipid contents without affecting the antidiabetic effect of RSG. Furthermore, we noticed that the expression of PPAR*γ* gene in hypothalamus was reduced by EA, while the expressions of leptin receptor and signal transducer and activator of transcription 3 (STAT3) were increased. Our results suggest that EA is an effective approach for inhibiting weight gain in T2DM rats treated by RSG. The possible mechanism might be through increased levels of leptin receptor and STAT3 and decreased PPAR*γ* expression, by which food intake of the rats was reduced and RSG-induced weight gain was inhibited.

## 1. Introduction

The thiazolidinedione (TZD) class of synthetic peroxisome proliferator-activated receptor*γ* (PPAR*γ*) agonists is used widely in diabetes to increase insulin sensitivity. Although the underlying mechanisms are complex and incompletely understood, induction of genes involved in glucose and lipid metabolism in macrophages, muscle, and adipose tissue is thought to play roles [1, 2]. However, in addition to enhancing insulin activation, TZDs induce weight gain in humans and rodent models by enhancing adipogenesis and fluid retention and increasing food intake [3, 4]. Weight gain may cause many medical consequences for type 2 diabetic patients, such as overweight or obesity, insulin resistance, and cardiovascular diseases. Few studies have investigated whether TZDs-induced weight gain can be abated, so studies are needed to explore whether the observed TZDs-related weight gain can be prevented by certain interventions.

Acupuncture, as one of the traditional medical approaches, has been used to treat obesity in China for many years. Several clinical trials have been carried out very recently [5–7], and evidences have supported the idea that acupuncture is an effective and safe approach for reducing body weight. Experimental studies are uncovering its mechanisms by which acupuncture could reduce food intake and increase energy consumption [8–10]. A recent study further demonstrated that EA stimulation increased peptide and mRNA levels of *α*-MSH and its precursors POMC and CART in the arcuate nucleus of hypothalamus (ARH) neurons to inhibit food intake [8]. Cabıoğlu and Ergene reported that EA therapy in obese women reduced serum total cholesterol, triglycerides, and LDL cholesterol levels [11], suggesting a direct or indirect role of EA in mobilizing energy storage. Based on those studies, questions are raised. Does EA stimulation protect weight gain caused by RSG administration in T2DM rats? What is the possible mechanism in CNS?

The nuclear receptor PPAR*γ* is a ligand-activated transcription factor that promotes adipogenesis and insulin sensitivity [12, 13], and it is expressed in several tissues and cell types including white and brown adipocytes, macrophages, skeletal muscle, liver, pancreatic *β*-cells [1, 14, 15]. The activation of PPAR*γ* is found to be a key factor of increased body weight when both diabetic mice and patients were treated with TZD [16–20]. In addition to its actions in peripheral tissues, PPAR*γ* plays a role in neuronal systems governing energy balance and insulin sensitivity. PPAR*γ* in hypothalamic neurons is involved in the control of energy balance, glucose metabolism, and autonomic function, suggesting that PPAR*γ* signaling in the CNS may affect energy intake and storage [21–23].

Recent evidence has proven that RSG, a new class of antidiabetic drugs, rather than reducing hyperglycemia and hyperinsulinemia in insulin-resistant states, inhibits leptin expression and its signal transduction in different cells and animal models [24–27]. Leptin, a peptide hormone mainly secreted by adipocytes, is a pleiotropic molecule that regulates food intake, hematopoiesis, inflammation, immunity, cell differentiation, and proliferation [28]. The effect of leptin on food intake is mediated in part via leptin receptors presented in the hypothalamus. Peripherally applied leptin in rodents induces a central signaling pathway that involves activation of signal transducer and activator of transcription 3 (STAT3) [29]. The requirement of this pathway to prevent severe hyperphagia and obesity was recently demonstrated in mice specifically lacking the STAT3-binding site of the leptin receptor [30] and in mice with reduced level of STAT3 protein selectively in the CNS [31]. After binding to the long leptin receptor (OBRb), STAT3 becomes phosphorylated by Janus kinase 2 (JAK2) and acts in the nucleus to regulate transcription [32].

In the present study, we employed T2DM rats as the animal model and treated them with RSG combined with EA on Zusanli (ST36) and Neiting (ST44) acupoints. Our study, for the first time, revealed that EA treatment is effective for inhibiting weight gain in T2DM rats treated with RSG. The possible mechanism might be through increased leptin receptor and STAT3 and decreased PPAR*γ* expressions in CNS, by which food intake of the rats was reduced and so that RSG-induced weight gain was inhibited.

## 2. Materials and Methods

### 2.1. Chemicals

STZ was purchased from Sigma Chemical Co. (St. Louis, MO). Rosiglitazone maleate tablets were obtained from Glaxo Smith Kline Pharmaceutical Group (Tianjin, China). The test kits for total cholesterol and triglyceride were purchased from Nanjing Jiancheng Bioengineering Institute (Nanjing, China); iodine [125I] insulin radioimmunoassay kit was purchased from Beijing North Biotechnology Institute (Beijing, China).

### 2.2. Animals

Male Sprague-Dawley rats, weighted at 100–110 g, were supplied by SLAC Laboratory Animal Company (Shanghai, China). The rats were housed under controlled conditions of temperature (22 ± 2°C) and relative humidity (50 ± 10%) with a 12 h light-dark cycle. They were allowed free access to standard rodent chow. This study was approved by Animal Ethics Committee of Nanjing University of Chinese Medicine (approval number: 16, date of approval: Jan 18, 2007), and all procedures were conducted in accordance with the guidelines of the National Institutes of Health Animal Care and Use Committee.

### 2.3. Induction of Diabetic Rats

The diabetic rats were induced according to Reed's method with minor modification [33]. Briefly, the rats were fed on high-fat diet (HFD). The HFD consisted of 15% lard, 5% sesame oil, 20% sucrose, 2.5% cholesterol, and 57.5% normal chow. After 4 weeks of dietary manipulation, T2DM rats were administrated with an intraperitoneally injection of STZ (35 mg/kg, dissolved in pH 4.5 citrate buffer). Control rats only received an equivalent volume of citrate buffer. Then experimental rats were continued on their original diets. Fasting blood glucose concentrations, body weight, and food intake were monitored once a week after STZ injection. Biochemical parameters (serum triglyceride, serum total cholesterol, and serum insulin) were measured on day 21 after vehicle or STZ injection. Rats in the T2DM group with nonfasting plasma glucose ≥16.67 mmol/L (300 mg/dL) were chosen as diabetic rats for further study [34].

### 2.4. Oral Glucose Tolerance Test

On day 22 after STZ or vehicle injection, oral glucose tolerance test (OGTT) was performed. Animals were fasted overnight and then received glucose solution (2 g/kg) orally. Blood samples were collected via the oculi chorioideae vein under light ether anaesthesia at 0 (just before administration), 15, 30, 60, and 120 min after glucose loading. Serum was separated by centrifugation. The concentrations of glucose and insulin in serum were measured using glucometer (Johnson & Johnson Biological Devices Co., Ltd., China) and insulin radioimmunoassay kit (North Institute of Biotech Co., Beijing, China), respectively. Homeostatic model assessment (HOMA) was used to assess the longitudinal changes in insulin resistance (HOMA-IR) [35].

### 2.5. Grouping of Animals and Parameters of Acupuncture

Normal diet rats were randomly divided into control group (Con) and electroacupuncture group (Con-EA). T2DM rats were randomly divided into T2DM group, T2DM-EA group, T2DM-RSG group, and T2DM-RSG-EA group. All of the rats were fed with normal diet. Rats in the EA group were physically restrained and then electroacupunctured on Zusanli (ST36) and Neiting (ST44), while rats in the control group were restrained in the same way, but without acupuncture. For the rats who received EA treatment, two acupuncture needles (Gauge-28, 0.16 mm) were separately inserted into each acupoint and an electrical current was provided to the needles through an electrical stimulator with parameters of 2/15 Hz at an intensity level of 1 mA (Han Acuten, WQ1002F, Beijing, China) for 30 minutes, once a day, six days a week. Rats in the RSG group orally received 5 mg/kg of RSG once a day, six days a week. Body weight and food consumption were monitored every week. After 6 weeks of EA or RSG treatment, all the rats were sacrificed with CO_2_ and samples were collected.

### 2.6. qPCR Analysis

Total RNA was isolated using TRIzol^®^ Reagent (Invitrogen, Cat# 15596-026) according to the manufacturer's recommendations. RNA concentrations were quantified and reverse-transcribed using ThermoScript^TM^ RT-PCR System for First-Strand cDNA Synthesis (Invitrogen, Cat# 11146-016). Gene expressions were detected using GoTaq qPCR Master Mix (Promega, Cat# A6001) in Strata gene MX3000P Real-Time PCR system (Genetimes, China). Relative gene expression levels were calculated by ΔΔCt and compared with GAPDH as internal control.

Primers were designed using Primer 5.0 software: PPAR*γ* (NM_001145366; forward: 5′-GTCACACTCTGACAGGAGCC-3′; reverse: 5′-CAC CGCTTCTTTCAAATCTTGT-3′), GAPDH (NM_017008; forward: 5′-AAGGGCTCATGACCACAGTC-3′; reverse: 5′-CAGGGATGATGTTCTGGGCA-3′), OBRb (AF287268; forward: 5′-TCCAGGTGAGGAGCAAGAG-3′; reverse: 5′-TTCAGCGTAGCGGTGATG-3′), and STAT3 (NM_012747; forward: 5′-TATCTTGGCCCTTTGGAATG-3′; reverse: 5′-GTTGTAGGACCATAGGGGTG-3′). Each reaction mixture (25 *μ*L total volume) contained 12.5 *μ*L Maxima SYBR Green Master mix (2X), 0.3 *μ*M of each primer, nuclease-free water, and 20 ng template DNA. Amplification of each gene was performed in duplicate runs and PCR conditions were 95°C for 10 min, followed by 40 cycles of 15 seconds at 95°C, 30 seconds at 60°C, and 30 seconds at 72°C.

### 2.7. Immunoblotting and Histological Analysis

Proteins were extracted from hypothalamus and the samples in SDS Loading Buffer were heated (100°C, 5 min), subjected to SDS-PAGE, transferred to PVDF or nitrocellulose membranes, and blocked (4°C, overnight) in PBST (PBS with 0.05% Tween 20) containing 5% nonfat dry milk or 5% BSA. Blots were incubated with a primary antibody in blocking buffer (overnight, 4°C) and then with a second antibody (1 : 1000~2000 dilution, 1 hr, RT). Signals were detected using SuperSignal ^*®*^ West Femto Maximum Sensitivity Substrate. Immunodetection of endogenous GAPDH was utilized to indicate that equivalent amounts of protein were present in samples added to the SDS PAGE (wells/lanes·*μ*g/L).

Rabbit anti-GAPDH (Cell Signaling, Cat#2118, 1 : 2000 dilution for Western blot); rabbit anti-PPAR*γ* for Western blot (Cell Signaling, Cat#2443, 1 : 1000 dilution for Western blot); rabbit anti-Stat3 (phospho Y705) for Western blot (Abcam, ab76315, 1 : 1000 dilution for Western blot); and rabbit anti-leptin (Abcam, ab3583, 1 : 1000 dilution for Western blot) were used.

#### 2.7.1. Histological Analysis

Adipose tissue were taken from all the animals and fixed for 24 hours with 10% formaldehyde in phosphate-buffered saline solution. Tissue pieces were washed with tap water, dehydrated in alcohol, and embedded in paraffin. Then, 5 mm sections were placed on glass slides and covered with saline. Hematoxylin-eosin (HE) staining was performed on the slides. HE staining was performed to assess adipocyte size of WAT. The tissue slides were incubated for 5 min in the hematoxylin solution. After water flushing and adding 0.5% ammonium hydroxide for 30 seconds, the tissue slides were put in 0.5% eosin for 2-minute dyeing and measured under microscope (Nikon TE2000, Japan).

### 2.8. Statistics

Data were presented as means ± standard deviation (SD). Statistics analysis was performed using SPSS 18.0; multiple group comparisons were made by ANOVA, and the comparison between two groups was determined using unpaired 2-tailed Student's *t*-test. *p* < 0.05 was considered statistically significant.

## 3. Results

### 3.1. Establishment of Diabetic Rat Model

**Figure 1:**
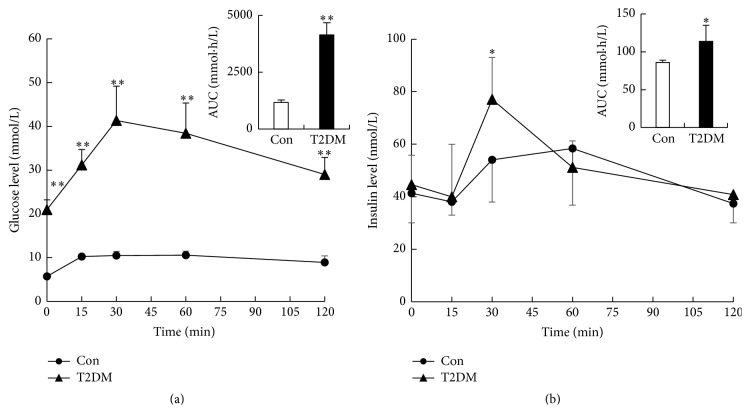
Serum glucose (a) and insulin (b) concentrations in T2DM and Con rats after oral glucose loading. Data were expressed as mean ± SD, *n* = 6 in Con, *n* = 26 in T2DM, and ^*∗*^ *p* < 0.05, ^*∗∗*^ *p* < 0.01 versus Con rats.

Biochemical parameters were measured in serum of the Con and T2DM rats (Table 1). It was found that levels of fasted blood glucose (FBG), triglyceride, and total cholesterol in serum of the T2DM rats were significantly higher than those in the Con rats, accompanied by reduction in body weight. T2DM rats also developed diabetic symptoms such as polyphagia, polyuria, and polydipsia. Higher level of serum insulin and severer HOMA-IR were observed in the T2DM rats. OGTT's result also showed that T2DM rats had significantly higher serum glucose and insulin concentrations induced by glucose loading, resulting in significant increase in AUC values of glucose and insulin (Figure 1). These indexes were similar to pathological state of type 2 diabetes, indicating the DM rats could be considered as type 2 diabetic rats [33, 34].

### 3.2. Electroacupuncture Treatment Significantly Reduced the Body Weight, Food Intake, and the Ratio of the Weights of WAT and BAT to Body Weight

**Figure 2:**
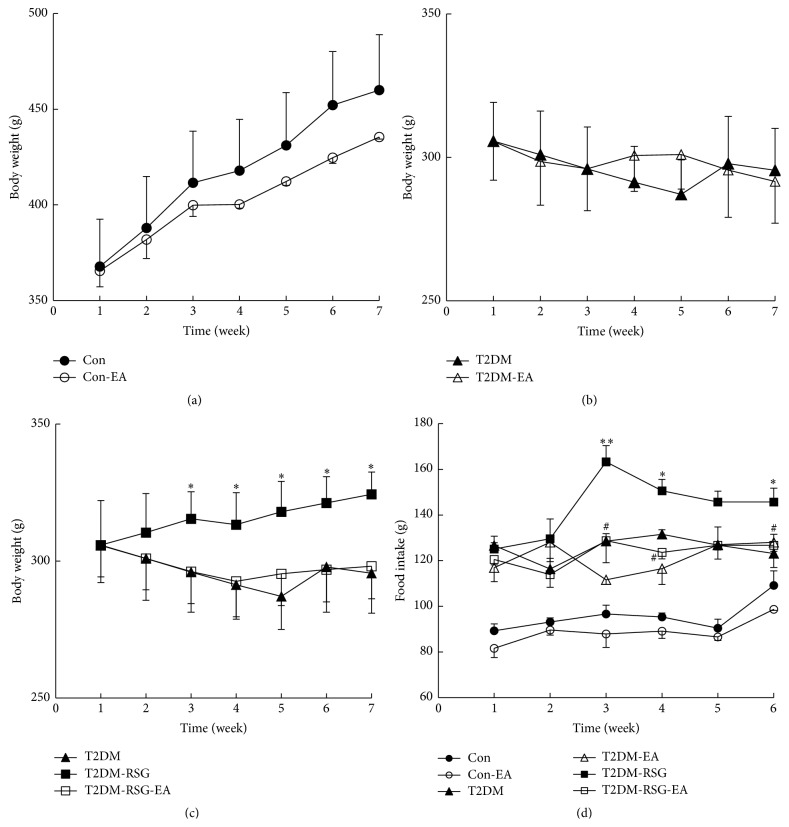
Effects of electroacupuncture and RSG treatment on each group. (a), (b), and (c) Observation of body weight (Con, Con-EA, T2DM, T2DM-EA, T2DM-RSG, and T2DM-RSG-EA groups), *n* = 7 in each group. (d) Measurement of food consumption, *n* = 7 in each group. Data were expressed as mean ± SD, ^*∗*^ *p* < 0.05, ^*∗∗*^ *p* < 0.01 versus T2DM rats, and ^#^ *p* < 0.05 versus T2DM-RSG rats.

In our study, electroacupuncture on Zusanli (ST36) and Neiting (ST44) markedly reduced body weight and food intake of the T2DM rats treated with RSG compared with the T2DM-RSG rats without acupuncture treatment (Figures 2(c) and 2(d)), whereas electroacupuncture did not significantly change body weight and food intake of Con and T2DM groups which did not receive RSG administration (Figures 2(a) and 2(b)), even after six weeks of intervention. For the first three weeks of treatment, rats in the T2DM-RSG group showed an increased appetite, which, however, had no notable fluctuation in the T2DM-RSG-EA group, suggesting that RSG may enhance appetite but electroacupuncture inhibited it to a certain extent in these rats.

**Figure 3:**
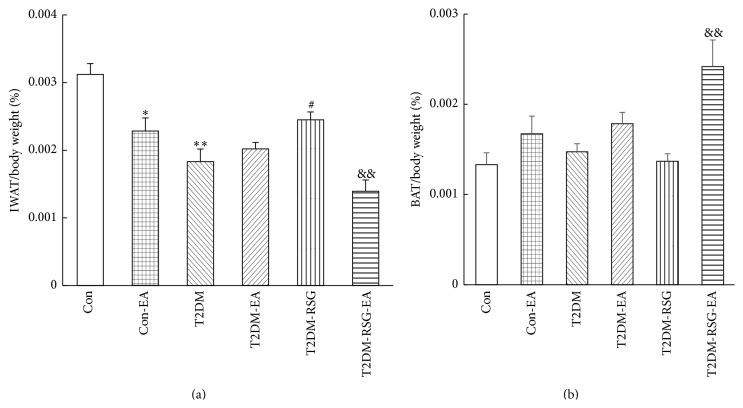
Effects of electroacupuncture and RSG treatment on (a) IWAT/body weight and (b) BAT/body weight. Data were expressed as mean ± SD, *n* = 6, ^*∗*^ *p* < 0.05, ^*∗∗*^ *p* < 0.01 versus Con rats, ^#^ *p* < 0.05 versus T2DM rats, and ^&&^ *p* < 0.01 versus T2DM-RSG rats.

**Figure 4:**
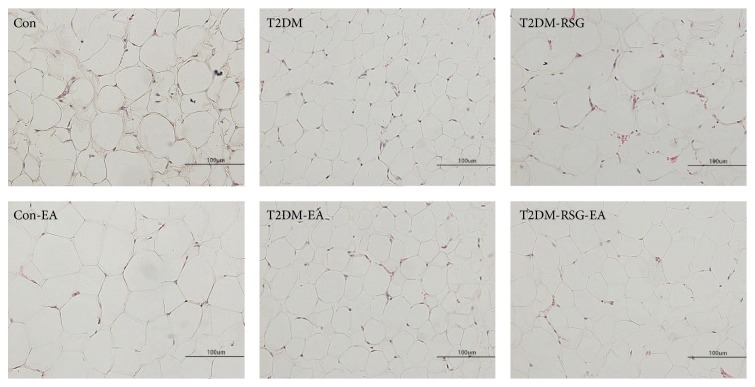
Histologic results of tissues stained with hematoxylin-eosin (HE) staining of each group after six weeks of treatment.

To evaluate the specific effect of EA on adipose tissue, we isolated the inguinal white adipose tissue (IWAT) from the rats in each group and calculated the ratio of IWAT to their individual body weight. We found that, in the T2DM-RSG rats, the ratio was higher than that in the T2DM rats. But after six weeks of treatment with electroacupuncture, the ratios were significantly decreased (Figure 3(a)). We analyzed the sizes of adipocytes by HE staining and observed obviously smaller size of the adipocytes of the IWAT in the T2DM-RSG-EA rats compared with that in the T2DM-RSG rats (Figure 4), suggesting that electroacupuncture might promote lipolysis in obese rats. In addition, after a six-week treatment with electroacupuncture, the ratio of BAT to body weight in rats of the T2DM-RSG-EA group was significantly higher than that in the T2DM-RSG group (Figure 3(b)). Our data suggest that the effectiveness of electroacupuncture on weight gain induced by RSG may be due to the decreased food intake and reduction in the ratio of IWAT to body weight.

### 3.3. Electroacupuncture Treatment Significantly Improved Metabolism of Cholesterol and Triglyceride in the Obese Rats

**Figure 5:**
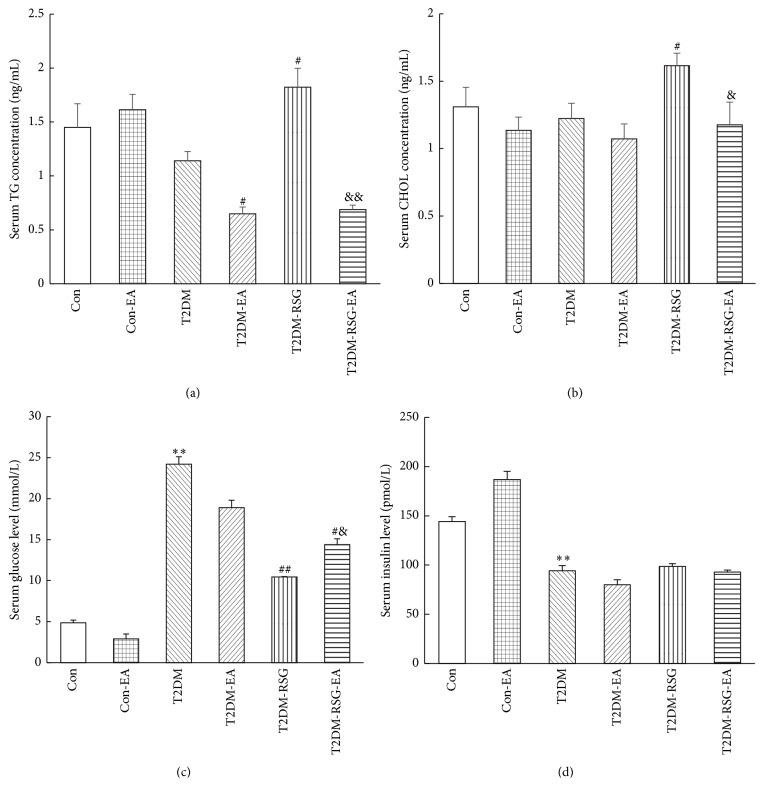
Serum triglyceride (a), total cholesterol (b), glucose (c), and insulin (d) concentrations were measured using a kit, as described in Section 2, respectively. Data were expressed as mean ± SD, *n* = 6, ^*∗∗*^ *p* < 0.01 versus Con rats, ^#^ *p* < 0.05, ^##^ *p* < 0.01 versus T2DM rats, and ^&^ *p* < 0.05, ^&&^ *p* < 0.01 versus T2DM-RSG rats. TG, triglyceride; CHOL, cholesterol.

We detected serum levels of cholesterol and triglyceride (TG) by using ELISA and found that, compared with the T2DM rats, cholesterol and TG levels were significantly elevated in T2DM-RSG rats after administration of RSG for six weeks; however, six weeks of treatment of RSG plus electroacupuncture completely reversed these levels to the normal levels (Figures 5(a) and 5(b)). FBG levels in the T2DM-RSG-EA rats were significantly decreased compared with the T2DM group (Figure 5(c)). Our data suggested that electroacupuncture effectively reduced the lipid contents in the T2DM rats treated with RSG, without affecting the antidiabetic effect of RSG.

### 3.4. Electroacupuncture Treatment Changed Ingestion-Related Gene Expression in CNS

**Figure 6:**
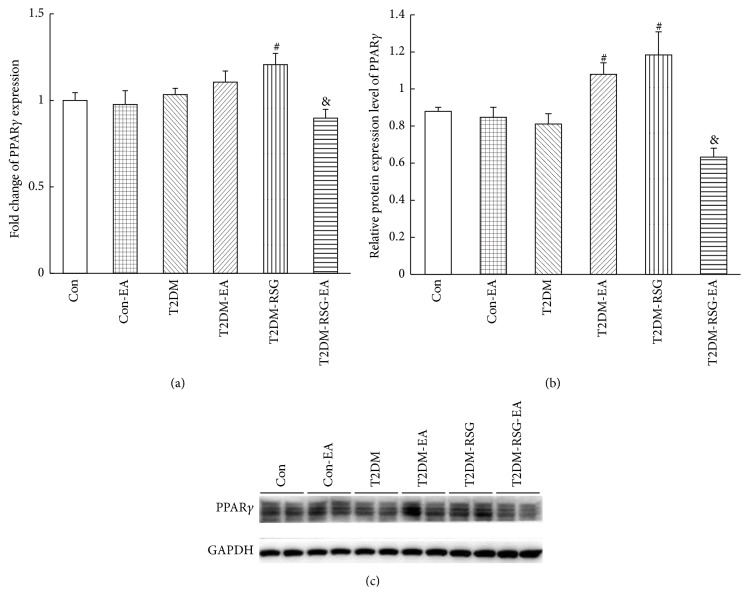
Effects of electroacupuncture and RSG treatment on PPAR*γ* in hypothalamus of each group. (a) mRNA of PPAR*γ* expression was detected by qPCR. Data were expressed as mean ± SD, *n* = 6, ^#^ *p* < 0.05 versus T2DM rats, and ^&^ *p* < 0.05 versus T2DM-RSG rats. (b) Quantitative analysis of PPAR*γ* protein. Data were expressed as mean ± SD, *n* = 6, ^#^ *p* < 0.05 versus T2DM rats, and ^&^ *p* < 0.05 versus T2DM-RSG rats. (c) Western blot result of PPAR*γ* protein in hypothalamus.

We found that the mRNA and protein expression of PPAR*γ* in hypothalamus of the T2DM-RSG rats increased than that in the T2DM rats, but not in the T2DM-RSG-EA group; furthermore, PPAR*γ* was obviously decreased after electroacupuncture treatment compared with the T2DM-RSG rats (Figures 6(a) and 6(b) and Table 2).

**Figure 7:**
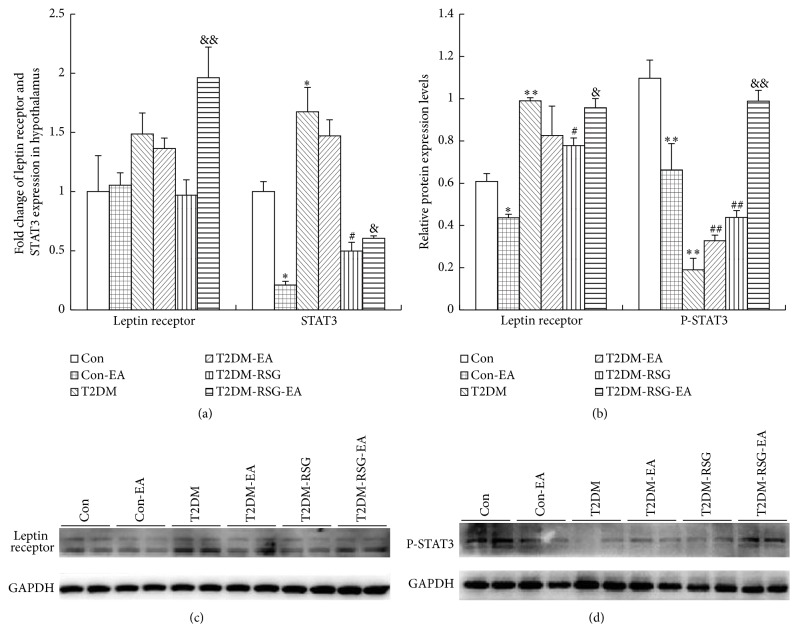
Leptin signaling expressions in hypothalamus of each group. (a) Representative mRNA of leptin signaling expressions was detected by qPCR. Data were expressed as mean ± SD, *n* = 6, ^*∗*^ *p* < 0.05 versus Con rats, ^#^ *p* < 0.05 versus T2DM rats, and ^&^ *p* < 0.05, ^&&^ *p* < 0.01 versus T2DM-RSG rats. (b) Quantitative analysis of leptin signaling proteins. Data were expressed as mean ± SD, *n* = 6, ^*∗*^ *p* < 0.05, ^*∗∗*^ *p* < 0.01 versus Con rats, ^#^ *p* < 0.05, ^##^ *p* < 0.01 versus T2DM rats, and ^&^ *p* < 0.05, ^&&^ *p* < 0.01 versus T2DM-RSG rats. (c) and (d) Representative Western blot results of leptin signaling proteins in hypothalamus.

To assess leptin responsiveness, we measured the expression of leptin receptor and phosphorylated STAT3, a downstream mediator of intracellular leptin signaling in hypothalamus. Our result shows that the protein expression of leptin receptor increased in the T2DM rats compared with the Con group though its mRNA level did not change and that, after being treated with RSG, the leptin receptor expression was significantly decreased, but EA combined with RSG treatment upregulated it again (Figures 7(a) and 7(b) and Table 2). Unexpectedly, the expression of leptin receptor decreased in hypothalamus in the control group treated with electroacupuncture (Con-EA). We then measured leptin-induced STAT3 phosphorylation in the hypothalamus. As indicated in Figure 7(b), leptin-induced P-STAT3 levels in the hypothalamus of RSG plus EA group were increased significantly compared with the T2DM-RSG rats. These data confirmed that electroacupuncture-inhibited food intake may be attributed to the activation of leptin-STAT3 pathway.

## 4. Discussion

Weight gain is commonly seen among patients treated with antidiabetic pharmacologic agents [36]. The thiazolidinediones (TZDs), rosiglitazone and pioglitazone, have also been associated with weight gain [37]. The 23-week trials with insulin sensitizing agents have shown consistent and dose-related weight gain ranging from 2.0 to 4.3 kg when used as monotherapy [38, 39]. Although conflicting results have been reported, the observed weight gain with TZDs is considered as a result of increased adipose tissue mass and water retention. TZDs are highly potential and selective agonists for PPAR*γ*, which is involved in the regulation of lipid and glucose metabolism [40, 41]. Activation of PPAR*γ* stimulates adipocyte fatty acid uptake, lipogenesis, and differentiation, so that results in weight gain. An adverse consequence of weight gain for patients with type 2 diabetes is overweight or obesity, which is linked to insulin resistance and other medical consequences such as cardiovascular disease. Current conventional therapeutic strategies, including caloric restriction, physical exercise, and drugs, however, can not effectively achieve adequate weight gain. Although exercise can result in short-term weight loss, only 5–10% of subjects can maintain the weight loss for more than a few years [42]. Acupuncture has been proved in animal models, such as obese rats, to reduce body weight [8, 9]. In the present study, electroacupuncture on Zusanli (ST36) and Neiting (ST44) markedly reduced body weight in the T2DM-RSG-EA rats and even in the control rats though the effect was mild (Figure 2). The ratio of the weight of IWAT, which is a major tissue for excess energy storage, to the body weight was significantly decreased upon acupuncture applications, suggesting that acupuncture can reduce energy storage to a certain extent. A decreased adipocyte size was observed in the T2DM-RSG-EA group (Figure 4), supporting its effect on inhibiting adipogenesis in the RSG-treated T2DM rats. Studies have shown that decreased appetite is one of the mechanisms by which acupuncture exerted weight loss effect in obese mice [8, 43]. Our present study also confirmed that electroacupuncture decreased food intake to a certain extent in the RSG-treated T2DM rats.

It is well known that weight gain effect of TZDs is a result of activated PPAR*γ* signaling on adipogenesis; however, TZDs also induce hyperphagia in rodent models, which directly linked to the weight gain adverse effect [44–46], suggesting a central site of action. Together with these previous reports, our study suggests that PPAR*γ* signaling in the CNS may influence energy intake and storage. PPAR*γ* is expressed in key brain areas, which controls energy homeostasis and glucose metabolism [47], raising the possibility that the CNS might be a previously unrecognized site for TZD action. Lu et al. showed important effects of brain PPAR*γ* on food intake, energy expenditure, and insulin sensitivity [48]. Deletion of brain PPAR*γ* led to both reduced food intake and increased energy expenditure. Our results revealed that RSG treatment increased PPAR*γ* expression in hypothalamus, and electroacupuncture deteriorated the raise of PPAR*γ* in RSG-treated T2DM rats. Our experiment, for the first time, provides the evidence that decreased PPAR*γ* by acupuncture in the CNS can regulate hyperphagia at least partially.

The effect of leptin, a major regulator of body weight and food intake [49], is particularly evident in rodents and humans lacking a functional form of the protein, resulting in severe obesity and greatly increased appetite [50, 51]. Treatment with recombinant leptin reversed the obese phenotype in leptin-deficient humans [52]. The effect of leptin on food intake is mediated in part via leptin receptors present in the hypothalamus. Peripherally applied leptin in rodents induces a central signaling pathway that involves activation of STAT3 [29]. The requirement of this pathway to prevent severe hyperphagia and obesity was recently demonstrated in mice specifically lacking the STAT3-binding site of the leptin receptor [30] and in mice with reduced levels of STAT3 proteins selectively in CNS [31]. After binding to the long leptin receptor, STAT3 becomes phosphorylated by Janus kinase 2 (JAK2) and acts in the nucleus to regulate transcription [32]. El-Haschimi et al. [53] showed that, in diet-induced obesity (DIO) mice, a classical mouse model of leptin resistance and obesity, recombinant leptin completely failed to induce STAT3 activation in hypothalamic extracts, demonstrating severe leptin-resistant signaling in the hypothalamus of DIO mice. This maybe relevant in the pathogenesis of insulin-resistant type 2 diabetes, which is often associated with overweight. In addition, microinjection of leptin into the arcuate nucleus [54], the ventromedial hypothalamus [54, 55], and the nucleus of the solitary tract [56] inhibited food intake. In our study, the protein expression of leptin receptor increased in T2DM rats compared with the control group though its gene level did not change. After treatment with RSG for six weeks, the leptin receptor expression was significantly decreased. After six weeks of treatment with EA on Zusanli (ST36) and Neiting (ST44), the rats with RSG treatment (T2DM-RSG-EA) displayed upregulated leptin receptor expression level. Leptin-induced P-STAT3 level in the hypothalamus of T2DM-RSG-EA group was increased significantly compared with that in the T2DM-RSG rats. These data confirmed that acupuncture may promote reduction of food intake through leptin-STAT3 signaling in the CNS.

Acupuncture is usually used to rectify the imbalance within the body under disease conditions. When the T2DM rats' body weight pathologically reduced, EA may incline to activate the translation of ingestion-related genes such as PPAR*γ* and OBRb to recover the abnormal phenotypes, including the reduced body weight. On the other hand, body weight of the T2DM rats treated with RSG was increased significantly compared with the T2DM group, and then EA may repress the expression of PPAR*γ* and OBRb genes to rectify the imbalance of metabolism, although the mechanisms need to be further studied. As for the unchanged gene expression but elevated protein level of PPAR*γ* and leptin receptor by EA applied on T2DM rats, some posttranscriptional regulation mechanism might be considered but need to be confirmed by further experimental studies in the future. Recent study reported that leptin signaling contributes to the metabolic features associated with breast cancer malignancy, such as switching the cells' energy balance from mitochondrial *β*-oxidation to the aerobic glycolytic pathway. Leptin binds to its receptor leading to JAK-mediated tyrosine phosphorylation of the intracellular domain and, through JAK-STAT and MAPK activation, induces phosphorylation and nuclear translocation of glucocorticoid receptor (GR). In the nucleus, GR binds to glucocorticoid-responsive elements in the Leptin gene, resulting in upregulation of leptin expression. Treatment with PPAR*γ* ligands blocks activation of JAK-STAT and MAPK signaling. PPAR*γ* also binds to GR and to glucocorticoid-responsive elements in LEP; formation of a GR-PPAR*γ* complex enables recruitment of nuclear receptor corepressors N-CoR1 and N-CoR2, which inhibit leptin transcription. Moreover, activation of PPAR*γ* also decreases leptin receptor expression and inhibits its transductional pathways [57]. Based on this literature and our present results, we propose that acupuncture may regulate PPAR*γ* expression in hypothalamus and then result in alteration of leptin expression, thus reducing food intake, although more biological experiments are needed to confirm the interaction of PPAR*γ* and leptin pathway in the CNS in the TZDs-treated diabetic models.

## 5. Conclusion

Taking together, our results, for the first time, provided experimental evidences that EA can inhibit weight gain side effect of RSG through increased level of leptin receptor and STAT3 and decreased PPAR*γ* expressions in CNS, which may contribute to reduced food intake. Further study for determining the interaction of PPAR*γ* and leptin-STAT3 pathway in the CNS of TZDs- treated T2DM rats is of profound significance.

## Figures and Tables

**Table 1:** Biochemical parameters in serum of the Con and T2DM rats.

Group	Initial body weight (g)	Final body weight (g)	Serum glucose (mmol/L)	Plasma triglyceride (mmol/L)	Plasma total cholesterol (mmol/L)	Serum insulin (pmol/L)	HOMA-IR
Con	142.33 ± 7.72	363.88 ± 22.95	4.82 ± 0.29	0.49 ± 0.07	1.24 ± 0.20	33.35 ± 3.68	42.02 ± 15.89
T2DM	139.16 ± 7.89	300.91 ± 22.26^*∗∗*^	18.97 ± 4.99^*∗∗*^	3.48 ± 1.95^*∗∗*^	1.96 ± 0.80^*∗∗*^	57.00 ± 16.00^*∗*^	10.42 ± 2.45^*∗∗*^

The T2DM rats were fed on high-fat diet for 4 weeks and then were given an intraperitoneally injection of STZ (35 mg/kg). Biochemical parameters (serum triglyceride, serum total cholesterol, and serum insulin) were measured on day 21 after vehicle or STZ injection. Each value is presented as mean ± SD (*n* = 6 in Con group and *n* = 26 in T2DM group). ^*∗*^ *p* < 0.05, ^*∗∗*^ *p* < 0.01 versus Con rats using Student's *t*-test.

**Table 2:** mRNA fold change of PPAR*γ*, leptin receptor, and STAT3 expression in each group.

Group	PPAR*γ*	Leptin receptor	STAT3
Con	1.00 ± 0.045	1.00 ± 0.303	1.00 ± 0.084
Con-EA	0.98 ± 0.079	1.05 ± 0.104	0.21 ± 0.031^*∗*^
T2DM	1.03 ± 0.036	1.49 ± 0.176	1.67 ± 0.207^*∗*^
T2DM-EA	1.11 ± 0.064	1.36 ± 0.088	1.47 ± 0.135
T2DM-RSG	1.21 ± 0.065^#^	0.97 ± 0.131	0.50 ± 0.073^#^
T2DM-RSG-EA	0.90 ± 0.050^&^	1.96 ± 0.258^&&^	0.61 ± 0.021^&^

Data were expressed as mean ± SD, *n* = 6, ^*∗*^ *p* < 0.05 versus Con rats, ^#^ *p* < 0.05 versus T2DM rats, and ^&^ *p* < 0.05, ^&&^ *p* < 0.05 versus T2DM-RSG rats using Student's *t*-test.
